# Gender Differences and Quality of Life in Parkinson’s Disease

**DOI:** 10.3390/ijerph18010198

**Published:** 2020-12-29

**Authors:** Pietro Crispino, Miriam Gino, Elena Barbagelata, Tiziana Ciarambino, Cecilia Politi, Immacolata Ambrosino, Rosalia Ragusa, Marina Marranzano, Antonio Biondi, Marco Vacante

**Affiliations:** 1 Internal Medicine Department, Lagonegro Hospital, 85042 Lagonegro (PZ), Italy; picrispino@libero.it; 2 Department of Internal Medicine, Rivoli Hospital, 10098 Rivoli (TO), Italy; miriam.gino@gmail.com; 3 Department of Internal Medicine, ASL 4 Chiavarese, Sestri Levante Hospital, 16039 Sestri Levante (GE), Italy; elenabarbagelata@yahoo.it; 4 Department of Medical, Surgical, Neurological, Metabolic and Geriatrics Sciences, Marcianise Hospital, ASL Caserta, University of Campania “L. Vanvitelli”, 81025 Naples, Italy; tiziana.ciarambino@gmail.com; 5 Department of Internal Medicine, Veneziale Hospital, 86170 Isernia, Italy; cecilia.politi@asrem.org; 6 ASL Bari, 70023 Bari, Italy; imma-ambrosino@libero.it; 7 Health Technology Assessment Committee, University Hospital G. Rodolico, 95123 Catania, Italy; ragusar@unict.it; 8 Department of Medical, Surgical and Advanced Sciences, University of Catania, 95123 Catania, Italy; marranz@unict.it; 9 Department of General Surgery and Medical-Surgical Specialties, University of Catania, 95123 Catania, Italy; abiondi@unict.it

**Keywords:** Parkinson’s disease, gender, dopamine, levodopa, quality of life

## Abstract

Parkinson’s disease has been found to significantly affect health-related quality of life. The gender differences of the health-related quality of life of subjects with Parkinson’s disease have been observed in a number of studies. These differences have been reported in terms of the age at onset, clinical manifestations, and response to therapy. In general, women with Parkinson’s disease showed more positive disease outcomes with regard to emotion processing, non-motor symptoms, and cognitive functions, although women report more Parkinson’s disease-related clinical manifestations. Female gender predicted poor physical functioning and socioemotional health-related quality of life, while male gender predicted the cognitive domain of health-related quality of life. Some studies reported gender differences in the association between health-related quality of life and non-motor symptoms. Depression and fatigue were the main causes of poorer health-related quality of life in women, even in the early stages of Parkinson’s disease. The aim of this review was to collect the best available evidence on gender differences in the development of Parkinson’s disease symptoms and health-related quality of life.

## 1. Introduction

Parkinson’s disease (PD) is a debilitating neurodegenerative disease that is responsible for cognitive impairment, non-motor, and motor symptoms, which has been found to significantly affect health-related quality of life (HRQoL). As in all chronic diseases, and especially PD patients, the psychological aspect has great importance to the quality of life. The World Health Organization (WHO) defined HRQoL as “individuals’ perception of their position in life in the context of the culture and value systems in which they live and in relation to their goals, expectations, standards and concerns” [1]. The assessment of HRQoL includes different aspects: motor and physical skills, mental health, somatic perception, and socioeconomic conditions [2,3].

The differences in the cognitive functions between men and women with PD have not been widely studied. Van Den Eeden and associates found an increased incidence of cognitive deficits, difficulties in the execution of activities of daily living (ADLs), a decrease of the fluidity of verbal production and facial expressions in men, while visual–spatial function impairment has been observed more frequently in women [4]. The symptoms of PD, including stiffness, behavioral and sleep disorders, especially in the REM phase, appear earlier and more often in men than women, while dyskinesia and depression are observed more frequently in women [5]. In general, female gender is associated with poorer scores in physical functioning and socioemotional HRQoL, while male gender mostly showed a faster decline in cognitive performances [6]. However, a prospective study carried out on PD patients observed poorer performances in all items for women, apart from self care, compared to men [7]. Another study reported a significant decline of life satisfaction (LS) in the second half of life only in male PD patients [8,9]. Some studies reported the existence of gender differences in PD patients with regard to HRQoL and non-motor symptoms (NMS). Fatigue and depression were the major causes of low HRQoL in women even in the early phases of PD [10].

HRQoL in PD patients is frequently assessed by the Parkinson’s Disease Questionnaire 39 (PDQ-39). The PDQ-39 includes eight HRQoL subgroups, which are generally summed up by a PDQ-39 summary score; the most important domains are cognition, physical-functioning, and socioemotional HRQoL. Other less frequently used tools, which are not specific for PD, include the Short Form (SF) 36, the EuroQoL (EQ-5D), the World Health Organization Quality of Life 100 (WHOQOL-100), and its shorter version, WHOQOL-BREF [11,12,13,14,15,16].

The aim of this review was to collect the best available evidence on the gender differences in the development of PD symptoms and HRQoL.

## 2. General Mechanisms Involved in Gender Differences in Parkinson’s Disease

### 2.1. Genetic Factors

There is a specific link between the gender and gene expression profiles of normal dopaminergic neurons, which underlie the predisposition of males for the development of PD [17]. The adaptive mechanisms of dopaminergic neurons that survived an injury are mediated by different processes in men and women. In a neurotypical brain, the genes involved in the pathogenesis of PD (i.e., α-synuclein and PINK-1) are upregulated in men, whereas the genes that are responsible for maturation and neuronal signal transduction are upregulated in women [17].

Changes in the expression of genes linked to proteolysis and the Wnt signal, and genes encoding for the protein kinase that controls activity in dopaminergic neurons were observed more frequently in women than men; on the other hand, major alterations in the expression of genes involved in binding to proteins and copper were found only in men [18,19].

According to Ji et al., a genetic mutation of the dopamine transporter (DAT) that differentially affects the nigrostriatal female and male transport systems could be responsible for the gender differences in the incidence and development of PD [20]. Furthermore, polymorphisms in the genes encoding for monoamine oxidase A (MAO-A) and monoamine oxidase B (MAO-B), which metabolize dopamine, could represent probable risk factors for PD [21], as well as polymorphisms in the dopamine D2 receptor (DRD2) gene that could predispose to PD [22].

Specific interactions between the gender, smoking habits and genetic polymorphisms of intron MAO-B 13 (allele G or A), MAO-A Eco RV (allele Y or N), and DRD2 Taq1B (allele B1 or B2) have been evaluated in individuals with idiopathic PD. The risk of developing PD in smokers was greater compared to non-smokers and men with genotype G. In women, the odds ratio was 0.62 for smokers compared to non-smokers with genotype GG/GA, and 0.64 for those with genotype A. These results suggested the existence of gender differences with regard to the modifying effect of the MAO-B genotype on the association of smoking with PD [22].

The gene sequence that encodes for Kinase 2, an enzyme-repeating protein rich in leucine (LRRK2), is frequently involved in PD [23,24]. However, only some variants of LRRK2 increased the risk of developing PD [23]. Many studies have been carried out on a hypothetical gender difference in the motor and non-motor symptoms associated with LRRK2 polymorphism. Indeed, PD patients with LRRK2 mutations are more likely to be women, without gender differences in motor and non-motor symptoms [24,25].

There is evidence that the genetic factors influencing inflammation could affect the risk of PD: San Luciano et al. investigated the polymorphisms for the promoter of the IL-6 gene and the beta estrogen receptor (ESR2), and showed that the GG genotype increased the risk of PD and had a different distribution between the sexes. Women with the AA genotype had a reduced risk of PD [26]. Another study found that the G genotype of the IL-6 gene, which influenced the expression of IL-6, was associated with a higher risk of PD, especially among men [27].

In summary, there is wide evidence of gender-based differences in the gene expression in dopaminergic neurons that may support the greater male susceptibility to the development of PD. Gender may influence the response to PD, indicating that the nature of PD and even the response to therapy may be gender-dependent [18].

### 2.2. Mitochondrial Function

Many neurodegenerative disorders are associated with mitochondrial dysfunction [28]. Alterations in the brain energy metabolism in PD are associated with an increase in oxidative stress and apoptosis, which lead to mitochondrial dysfunction, and affect calcium homeostasis, and, in turn, brain metabolism [29]. Therefore, it has been hypothesized that mitochondria could be involved in the pathogenesis and progression of PD [30].

Weiduschat et al. observed an increase of high-energy brain substrates in males, especially in the striatum of the hemisphere affected by PD. Furthermore, they observed a 16% reduction in ATP and a 12% reduction in phosphoric energy substrates in the gray matter of men, suggesting a greater impact of mitochondrial dysfunction in men than in women [29].

The level of mitochondrial hemoglobin in the neurons is reduced in subjects who develop PD [31]. The localization of hemoglobin in the mitochondrial compartment could represent a protective mechanism in circulating leukocytes and neurons [32]. Indeed, the different hemoglobin modulation between cytosol and mitochondria could be the consequence of the different bioavailability of oxygen between the two genders; females are more predisposed to anemia and are physiologically lower iron levels than males, and this could play an important role in neurodegeneration [30,33].

Many therapeutic approaches focused on a potential mitochondrial etiology of PD, for example mitochondrial enhancers (i.e., coenzyme Q10, and vitamin K2), kinetin triphosphate (KTP), and selective MAO-B inhibitors such as selegiline and rasagiline. However, the results have been controversial so far, and further clinical trials targeting mitochondrial dysfunction, and aiming to reduce disability and improve HRQoL are needed [34].

### 2.3. Inflammatory Response

Some studies have been carried out on animal models in order to evaluate whether there are gender differences in the inflammatory response that could underlie the production of free radicals, thus affecting the degeneration of the nigro-striatal dopaminergic pathway. It is well known that rotenone, a broad-spectrum pesticide, could promote the production of free radicals by inhibiting the transport chain of mitochondrial electrons and leading to the cell death of dopaminergic neurons in the substantia nigra [35]. Dopaminergic neurons are mostly vulnerable to free radicals due to their reduced antioxidant activity, which is associated with an increase in iron deposits [36].

Mitra et al. studied the main brain regions involved during the pathogenesis of PD in both sexes in a murine model; they observed that, under non-pathogenic conditions, the activity of enzymes against free radicals, such as catalase, glutathione S-transferase (GST), and superoxide dismutase (SOD) varied between brain regions and between genders. The number of 3,4-dihydroxyphenylalanine (DOPA) decarboxylase positive cells was similar in both male and female animals, and the level of the proinflammatory cytokine TNF-α was similar in the frontal cortex and hippocampus in both genders, except for the substantia nigra. Furthermore, during treatment with rotenone, the activity of enzymes against free radicals increased in both genders. Astrocytes and microglial cells were raised in both genders, except for in the substantia nigra of males, in which the density of the astrocytes was unchanged, and was correlated with a reduction in the rate of microglial cells. After treatment with rotenone, there was an increase in TNF-α in the frontal cortex and substantia nigra of both genders, whereas the binding for estrogens and nuclear-cytosolic receptor α and β was different in the brain regions of the two genders [37].

There is evidence that the modulation of chronic inflammation implicated in PD could contribute to delay neurodegeneration, thus changing the course of the disease. Lifestyle modifications (such as the adoption of a Mediterranean diet and physical activity) could attenuate the inflammatory state, with beneficial effects on PD clinical manifestations and HRQoL [38,39,40].

## 3. Clinical Features

Haaxma et al. found specific differences in the clinical manifestations of PD between genders [41]. The ratio between men and women is about 2:1 at the time of diagnosis; women are older than men (53.4 vs. 51.3 years), and are more expected to show tremors as an initial symptom versus stiffness or bradykinesia. It is noteworthy that women could show fewer symptoms in the preclinical stage of PD compared to men, perhaps due to the protective role of estrogens. Haaxma et al. showed that the action of estrogens can be related to different iron levels in men and women [41]. Once PD has reached a more advanced and established clinical stage, no more gender differences can be observed; indeed, estrogens may play a crucial role in neuroprotection against degenerative damage, but do not offer any advantage after the beginning of clinical symptoms [41].

Using single photon emission computed tomography (SPECT) neuroimaging, higher levels of dopamine were observed in the nigrostriatal nerve endings of women compared to men at the onset of PD symptoms. The repercussions of gender differences on the efficiency of nerve endings in the nigro-striatal pathway of subjects with PD remain unclear, although some studies have reported better dopaminergic regulation at the striatal level in women compared to men, but no conclusive explanation of the underlying mechanisms has been found [42,43,44].

Scott et al. reported that, at the beginning of the disease, men listed more symptoms that were suggestive of PD, and women showed more anxiety-related symptoms that were referable to decreased mood, suggesting gender differences in the signaling and awareness of symptoms [45]. Other studies evaluated the gender differences in the ADLs in PD patients with clinical symptoms [46,47,48]. A study analyzed the gender differences after thalamotomy, pallidotomy, and deep brain stimulation, showing fewer preoperative scores in ADL assessment, but with post-operative mirroring between the two sexes. Women were more compromised and had a longer duration of illness compared to men. This suggested that there was a propensity to postpone invasive care procedures by women, or perhaps that women coped better with their symptoms [46]. Accolla et al. assessed the relationship between the clinical features and ADLs of PD patients before and after deep brain stimulation without showing gender differences in the length or severity of PD, or in the presence of major symptoms such as tremor or stiffness, although women showed more severe dyskinesia and were less responsive to bradykinesia drugs. After a one year follow up, a significant resistance to treatment was observed in women compared to men, and only women showed better performances in ADLs, albeit not significantly [47]. Baba et al. showed that many PD patients did not differ by gender, in terms of the mean age at onset, length of disease, progression rate, or early symptoms, whereas—after onset—women showed more impairment than men in depression, ADLs, and decline in cognitive abilities [48].

A higher prevalence of levodopa-induced dyskinesia has been reported in women versus men with PD. Indeed, Zappia et al. pointed out that women with less body weight received a higher dosage of levodopa per kilogram of body weight and more frequently showed dyskinesia compared to men [49], whereas Accolla et al. showed that women with low body weight who received an increasing dosage of levodopa experienced a worsening of dyskinesia that was probably induced by the drug itself [47].

Sleep disturbances are also frequent in patients with PD, and many studies have reported gender differences in this topic. Two studies carried out on patients with Rapid Eye Movement (REM) sleep behavior disorders showed a different onset in men and women, especially in patients with dementia traits [50,51]. From a behavioral point of view, a study by Fernandez et al. showed that men with PD showed more traits of physical and verbal aggression and inappropriate behavior, while women showed a marked mood deflection [52].

Studies have showed that PD-related HRQoL factors—such as the grade of motor impairment, the general severity, and the levodopa equivalent daily dosage—affected HRQoL less significantly than non-motor symptoms [53,54,55,56]. However, the relationships between the clinical features of PD and HRQoL may differ significantly based on the assessment instruments. For instance, depression, which is a common comorbidity, is poorly assessed through the EQ-5D, but not through the PDQ-39 and the WHOQOL-100/WHOQOL-BREF. On the contrary, motor deficits are underrated through the PDQ-39 and the WHOQOL-100/WHOQOL-BREF, and correlated with HRQoL in the EQ-5D [7].

## 4. Cognitive Status

Cognitive decline is more pronounced in patients with PD compared to the general population, and recent studies have reported that around 80% of patients with PD will suffer from dementia after twenty years of illness [57]. The diagnosis of dementia in PD patients raises the burden of caregivers significantly, and reduces HRQoL. Moreover, mild cognitive impairment (MCI) could aggravate disability, and general functional impairment proceeds together with cognitive decline [58]. Cognitive impairment may impair ADLs and decrease task-oriented coping in PD patients, resulting in poorer HRQoL [59,60]. A longitudinal study carried out on a PD cohort assessed the impact of cognitive modifications on HRQoL, and showed that impairment in attention affected HRQoL significantly.

MCI in PD was correlated with poorer HRoQL over a 3-year follow up, and cognitive impairment affected HRQoL in patients who developed dementia over the follow-up period [61]. Cereda et al. studied the association between dementia and gender in PD patients, and found a greater prevalence of dementia after the age of 65 in women than in the general population, in which dementia prevailed in men instead [62]. Pasotti et al. reported that women had better scores in long-term memory tests and men showed better visuospatial skills, but these differences decreased and disappeared as the disease progressed [63].

Studies on cognitive changes in PD have investigated the activity of the basal ganglia [64,65,66,67,68], prefrontal cortex [69,70,71,72], fronto-striatal areas [73,74,75], or cortico-striatal areas (frontal and parietal) [76,77,78,79,80]. Riedel et al. [81] did not detect significant differences between the genders in the total Mini-Mental State Examination (MMSE) [82] or Parkinson Neuropsychometric Dementia Assessment (PANDA) score [83] in PD, although women scored significantly worse than men in motor severity stages. As the MMSE and the total score of the PANDA were used to assess general cognitive state and the outcomes were not compared with controls, it was difficult to detect changes in specific cognitive abilities.

Data from studies by Locascio et al. [84], Clark et al. [85], and Davidsdottir et al. [86] suggested that gender could influence cognitive impairment in subjects with PD. Gender differences emerged in the Road Map Test of Direction Sense, highlighting better skills in men for right-to-left discrimination and the cognition of space, and better scores for verbal fluency tests for women. At the follow up, the performance of men decreased more rapidly in the course of PD compared to women [87]. A study by Davidsdottir et al. reported that the same rate of men and women showed no less than one problem related to visual or visual-spatial performance. The study also demonstrated a relationship between gender and the side of PD onset with regard to spatial abilities.

In fact, men who manifested PD on the left side reported greater difficulties in assessing spatial relationships than women with the same form of the disease; on the contrary, no gender differences were observed in patients with a PD form on the right side. These results correlate the side of the onset of symptoms with low levels of dopamine in the contralateral hemisphere [86]. Other studies reported a deficiency in the expression of facial emotions, particularly irritation and surprise, and lack in the manifestations of fear in men.

Women instead showed more interpersonal problems [85]. These results are in accordance with those of previous research showing that women with PD had lower levels of HRQoL [88] and more symptoms of depression than men [47,48,52,81].

Davidsdottir et al. analyzed spatial navigation capacity and visuospatial abilities, contrast sensitivity and perception of movement, perception of optical flux and visual dependence [86]. Patients with PD of the left hemisphere were commonly more vision-dependent than patients with contralateral PD, who were more vision-dependent compared to controls. Other studies did not find differences in on visuospatial functioning between men and women with PD [77,78,89,90].

Cronin-Golomb and Braun reviewed the relationship between gender differences and the side of onset, defining PD as a disconnection syndrome [79,89]. Overall, these studies suggested that gender differences could affect only specific visuospatial skills, and could correlate with lateral manifestations of PD.

## 5. Motor Functions

There is evidence that gender could influence the manifestations and the grade of neuromotor symptoms in PD. Solla et al. evaluated the sexual disparities in motor symptoms among Sardinian individuals with idiopathic PD using the Unified Parkinson’s Disease Rating Scale (UPDRS), and observed that women are more likely to have poorer motor instability and tremors as an early symptom of PD, and have a lower UPDRS instability score compared to men [91].

The severity of motor disorders, dosage of levodopa, younger age at diagnosis and female gender are significant risk factors for motor fluctuations [92]. Haaxma et al. showed that women had tremors more frequently than men, both as initial symptoms and in the course of PD [41]. Lubomski et al. studied motor symptoms in 129 men and 81 women with PD, and discovered that men had more severe disease and more pronounced motor disorders [93]. Szewczyk-Krolikowski et al. studied sexual disparities in motor symptoms among 490 PD patients within three years of diagnosis. The authors observed that there was greater severity and greater modifications of symmetry in the face, neck, and arms in men compared to in women, and more postural difficulties in women [94]. Song et al. did not observe significant differences in the UPDRS total scores between the genders, whereas no significant differences between men and women were reported in the scores for primary and finer motor functions [95]. Motor symptoms may significantly affect HRQoL in PD patients, and improving this feature could be crucial for an optimal management of the disease in both genders [96].

## 6. Mood Symptoms

A cross-sectional study of 569 drug-naïve PD patients showed that female gender, length of disease, UPDRS III score, depression, and Non-Motor Symptoms Scale (NMSS) subscores such as attention/memory, mood/apathy, sleep/fatigue, and gastrointestinal scores affected the HRQoL in individuals with drug-naïve PD [97]. A systematic review by Soh et al. underlined the importance of depressive symptoms for HRQoL factors in PD patients [53]. Depression may also affect HRQoL by worsening disability and cognitive function, which represent negative HRQoL factors [58,98,99].

According to Solla et al., women had higher scores on the NMSS, including severe sleep difficulties, increased fatigue, and mood disorders such as apathy, anxiety, sadness, depression, and lack of motivation, whereas men reported higher sexual dysfunction levels [91]. Depression is a very common NMS in PD patients, with a prevalence of around 40% and a significant negative impact on HRQoL [100]. Some studies showed no gender differences in the development of depression in PD [101,102], while others observed a higher risk of developing depression in women [103].

A study evaluated the prevalence and severity of NMS related to PD without finding significant gender differences in the prevalence of fatigue, nervousness, sadness, restless leg syndrome, constipation, pain sensitivity, weight modifications, loss of taste or smell, and increased sweating; however, these symptoms were more severe in women. The same study showed that men had more severe sexual dysfunction and daytime sleepiness [104].

Guo et al. showed that patients with late-onset PD had more NMS and higher NMSS scores compared to patients with early-onset PD, but they did not find any gender difference [105]. Other studies reported an improvement of mood symptoms after therapy in both genders, although men had a greater risk of developing urinary disorders due to dopaminergic treatment [106].

In conclusion, mood symptoms could represent potential predictors of HRQoL in PD. It has been observed that excessive daytime sleepiness, sleep disturbances, anxiety and depression are common in PD patients, and are significantly associated with each other [107].

## 7. REM Phase of Sleep

REM sleep behavior disturbances are frequently observed in PD patients, and may have a considerable impact on the HRQoL. The disorder is characterized by a lack of muscular atony during REM sleep; some studies observed an incidence of the disorder in 43% of men and 31% of women with PD [108,109,110]. Postuma et al. reported that men with violent nocturnal behaviors could show these symptoms either as idiopathic or linked to a neurodegenerative disorder, while women with REM sleep disturbances mainly suffered from a PD-related neurodegenerative disorder [111]. A study by Bjørnarå et al. did not report significant differences in the frequency of probable REM sleep behavior disorders among men and women with PD. Nonetheless, women reported significantly fewer fights and aggressive behavior during dreams, but had more disturbed sleep compared to men [110].

## 8. Response to Treatment

Levodopa represents the gold standard of symptomatic efficacy in the drug treatment of PD. Wearing-off (WO) is a complication that can occur in patients with PD after long-term levodopa therapy. During WO, symptoms of PD begin to return or aggravate before the administration of the next dose of levodopa, and improve after the administration of the next dose [112]. A study reported that women with PD had an 80% increased risk of WO compared to men for both motor and NMS scores [113]. Another study observed that bilateral subthalamic nucleus deep brain stimulation (STN DBS) had similar efficacy in both men and women as a therapy for the motor complications of PD, and for improving HRQoL [114].

During the progression of PD, women frequently showed more benign clinical characteristics, suggesting the potential favorable effects of estrogen [115,116,117]. Women enrolled in the Parkinson’s Disease on Estrogen Therapy Replacement in the Menopause Years (POETRY) study showed improved scores on the UPDRS. Therefore, it has been suggested that estrogen replacement therapy (ERT) could improve PD symptoms, and could represent a chance to reduce the dosage of levodopa in women [118]. A recent meta-analysis showed that ERT significantly reduced the risk of the onset and progression of PD (Odds ratio (OR) 0.470, 95% confidence interval (C.I.) 0.368 to 0.600, *p* < 0.001) compared with the control group [119]. Another meta-analysis reported no association between menopausal hormone therapy and PD (OR 1.14, 95% C.I. 0.95 to 1.38, I2: 65%); however, a subgroup analysis highlighted a significant correlation between PD and progestogen (OR 3.41, 95% C.I. 1.23 to 9.46) or combined estrogen–progestogen use (OR 1.49, 95% C.I. 1.34 to 1.65). This relationship was determined by the length of exposure (Coef1 = 0.0626, p1 = 0.04) [120].

Further studies are needed in order to evaluate the full effects of PD treatments and their implications for HRQoL [121,122].

## 9. Conclusions

Many studies have reported gender differences in HRQoL in patients with PD. The progression of symptoms in PD is delayed in women by elevated levels of physiological dopamine at the nigro-striatal level due to estrogen activity [41]. A preventative role of estrogens has been suggested due to their anti-inflammatory properties, but larger studies are needed in different populations in order to assess the association between inflammatory genes and the effects of estrogens [123].

Among patients with PD, women suffered more frequently from depression and anxiety, whereas in men, tremors were more often associated with mild motor impairment and more significant striatal degeneration, resulting in greater disability and a higher frequency of WO [6].

The motor symptoms that mostly affected the overall life quality were gait impairment and complications due to treatment [124]. In order to reduce the impact of PD on HRQoL, it should be crucial to consider the level to which demographic factors, motor symptoms and NMS contribute to quality of life [54].

Motor symptoms and NMS are significant and independent causes of poor HRQoL in patients with PD, and a number of studies have shown that depression is the strongest determinant of low HRQoL [125]. Overall, women with PD showed more positive outcomes with regard to non-motor symptoms, cognitive functioning and emotion processing, even if they reported a more severe experience of PD-related clinical symptoms [126]. Further studies are needed in order to assess the role of gender differences in the development of cognition, motor, and non-motor symptoms of PD, and how they could affect HRQoL.
